# Treatment Effectiveness of Venetoclax‐Based Therapy After Bruton Tyrosine Kinase Inhibitors in Chronic Lymphocytic Leukemia: An International Real‐World Study

**DOI:** 10.1002/ajh.27563

**Published:** 2024-12-19

**Authors:** Nilanjan Ghosh, Toby A. Eyre, Jennifer R. Brown, Nicole Lamanna, Beenish S. Manzoor, Catherine C. Coombs, Hande H. Tuncer, Chaitra Ujjani, Lori A. Leslie, Lindsey E. Roeker, Matthew S. Davids, Joanna M. Rhodes, Alan P. Skarbnik, Wendy Sinai, Isabelle Fleury, Brian T. Hill, Nicolas Martinez‐Calle, Paul M. Barr, Dureshahwar Jawaid, Nnadozie Emechebe, Laurie Pearson, Frederick Lansigan, Yun Choi, Christopher E. Jensen, Bita Fakhri, Deborah M. Stephens, Steven E. Marx, Stephen J. Schuster, Michael Coyle, Irina Pivneva, Talissa Watson, Annie Guerin, Mazyar Shadman

**Affiliations:** ^1^ Levine Cancer Institute, Atrium Health, Wake Forest University School of Medicine Charlotte North Carolina USA; ^2^ Churchill Hospital Oxford University Oxford UK; ^3^ Dana‐Farber Cancer Institute Boston Massachusetts USA; ^4^ Columbia University New York New York USA; ^5^ AbbVie Inc. North Chicago Illinois USA; ^6^ University of California Irvine Irvine California USA; ^7^ Fred Hutchinson Cancer Research Center Seattle Washington State USA; ^8^ John Theurer Cancer Center, Hackensack Meridian Health Hackensack New Jersey USA; ^9^ CLL Program, Leukemia Service, Division of Hematologic Oncology Memorial Sloan Kettering Cancer Center New York New York USA; ^10^ Rutgers Cancer Institute of New Jersey New Brunswick New Jersey USA; ^11^ Lymphoma and CLL Program, Novant Health Cancer Institute Charlotte North Carolina USA; ^12^ Hôpital Maisonneuve‐Rosemont, University of Montreal Canada; ^13^ Cleveland Clinic Cleveland Ohio USA; ^14^ Nottingham University Hospitals, NHS Trust Nottingham UK; ^15^ Wilmot Cancer Institute, University of Rochester Medical Center Rochester New York USA; ^16^ University of Massachusetts Worcester Massachusetts USA; ^17^ Dartmouth Cancer Center Lebanon New Hampshire USA; ^18^ Tufts Medical Center Boston Massachusetts USA; ^19^ Lineberger Comprehensive Cancer Center, University of North Carolina at Chapel Hill Chapel Hill North Carolina USA; ^20^ Stanford Cancer Institute, Stanford University Stanford California USA; ^21^ University of Pennsylvania Philadelphia Pennsylvania USA; ^22^ Lowell General Hospital Lowell Massachusetts USA; ^23^ Analysis Group Inc. Montreal Quebec Canada

**Keywords:** Bruton tyrosine kinase inhibitor, chronic lymphocytic leukemia, real‐world outcomes, venetoclax

To the Editor,

The treatment landscape of chronic lymphocytic leukemia (CLL)/small lymphocytic lymphoma (SLL) has evolved drastically with the introduction of targeted agents, including covalent Bruton tyrosine kinase inhibitors (cBTKis) and B‐cell lymphoma 2 inhibitors (BCL2is) [1]. However, the development of cBTKi resistance (leading to disease progression) and intolerance due to adverse events limits durable cBTKi efficacy [1].

Venetoclax‐based therapy, including fixed‐treatment‐duration combinations, has demonstrated improved and sustained efficacy and manageable toxicity versus chemotherapy/chemoimmunotherapy (CT/CIT) for patients with previously untreated or relapsed/refractory CLL in phase 3 clinical trials [1]. Because cBTKis were not yet or were just recently approved during patient recruitment for the initial venetoclax landmark clinical trials, there were few patients with previous exposure to cBTKis and, thus, limited evidence supporting venetoclax efficacy in these settings [2].

Although clinical trials like M14‐032 have established the clinical benefit of venetoclax after cBTKis, real‐world studies report on small subgroups of patients with CLL or patients who received venetoclax primarily in later lines of therapy, following CT/CIT [2, 3, 4, 5]. Additionally, there is limited literature evaluating real‐world venetoclax outcomes among patients stratified by reasons for cBTKi discontinuation [3], venetoclax‐based regimen (particularly, VR combination) [4, 5], or prior CT/CIT exposure. This study assessed real‐world clinical outcomes of patients with CLL/SLL, who received venetoclax‐based therapy after cBTKi therapy, overall and stratified by line of therapy, prior CT/CIT exposure, discontinuation of cBTKi therapy due to intolerance or disease progression, and for treatment with venetoclax + rituximab (VR).

Data for the current analysis (06/01/2018–12/27/2023) were collected as part of the CLL Collaborative Study of Real‐World Evidence (CORE; Supporting Information). Adult patients diagnosed with CLL/SLL were included if they initiated a first‐line (1 L) or new line of therapy in a relapsed/refractory setting after 02/12/2014 (US approval date of ibrutinib for CLL/SLL) and if they received a venetoclax‐based therapy (i.e., monotherapy or combination therapy with rituximab or obinutuzumab) following discontinuation of a cBTKi‐based regimen.

Clinical outcomes assessed following initiation of venetoclax‐based therapy included overall response rate (ORR), time to next treatment or death (TTNT‐D), and progression‐free survival (PFS). Outcomes were assessed for the overall population and stratified by line of therapy (1 L cBTKi➔second‐line [2 L] venetoclax; 2 L cBTKi➔third‐line [3 L] venetoclax), CT/CIT exposure before cBTKi treatment for 2 L cBTKi➔3 L venetoclax, primary reason for cBTKi discontinuation (either intolerance [D_I_ group] or progression [D_P_ group]), and VR treatment. Additional details are provided in Supporting Information.

Among the 2293 patients in CORE, 205 received venetoclax after cBTKi and were included in the analyses (Figure S1). The median (IQR) age at venetoclax initiation was 68.7 (62.2, 76.4) years and 68.8% were male (Table S1). Of patients with documented genetic characterization, 69.6% (48/69) had unmutated IGHV (D_I_: 63.0% [17/27], D_P_: 73.1% [19/26], VR: 69.6% [16/23]) and 24.2% (30/124) had 17p deletion or TP53 mutation (D_I_: 14.3% [7/49], D_P_: 34.6% [18/52], VR: 17.4% [8/46]) at venetoclax‐based therapy initiation. The most common comorbidities were cardiovascular (46.8%) and endocrine/metabolic (23.4%) conditions. Additional patient characteristics are provided in Supporting Information.

Overall, 123 patients initiated venetoclax monotherapy (60.0%), 64 initiated VR (31.2%), and 18 initiated venetoclax + obinutuzumab (VO; 8.8%; Table S2). Seventy‐one patients (34.6%) initiated venetoclax‐based therapy in the 2 L (i.e., 1 L cBTKi➔2 L venetoclax group; VR: 31/71 [43.7%]), while 73 (35.6%) did so in the 3 L (i.e., 2 L cBTKi➔3 L venetoclax group; VR: 23/73 [31.5%]); the remaining 61 initiated venetoclax in the fourth line or later. A median (IQR) of 2 (1, 3) prior lines of therapy was received among all patients. The median (IQR) treatment duration with venetoclax was 14.4 (3.9, 28.0) months (monotherapy: 14.1 [3.7, 30.1]; VR: 16.4 [7.17, 28.2], VO: 6.3 [2.6, 16.5]), and 80.0% maintained the same dose through the treatment; only 18.0% of patients required a dose reduction. The median (IQR) follow‐up after venetoclax‐based therapy initiation was 16.5 (6.0, 30.5) months. Additional treatment characteristics are provided in Supporting Information.

For all patients who initiated venetoclax‐based therapy, the median (IQR) treatment duration for the prior cBTKi was 20.2 (8.4, 39.2) months, and the most common reasons for discontinuation included intolerance (42.9%) and disease progression (37.1%, Table S2). The prior cBTKi treatment regimens received included ibrutinib (85.4%), acalabrutinib (6.8%), and ibrutinib + rituximab (2.9%). Among the 73 patients who initiated venetoclax‐based therapy as 2 L cBTKi➔3 L venetoclax, 1 L treatment regimens prior to cBTKi included CT/CIT (86.3%), targeted agents (11.0%; i.e., ibrutinib: 6.9%; other: 4.1%), and rituximab (2.7%).

Clinical outcomes are presented in Table 1, Figure S2 (ORR), Figure S3, (PFS), and Figure S4 (TTNT‐D).

**TABLE 1:** Clinical outcomes for the overall sample and stratified by line of therapy, discontinuation of cBTKi therapy due to intolerance or disease progression, and treatment with VR.

		Response		PFS^a^				TTNT‐D^b^			
		*N*	ORR^c^	*N*	Median (CI), months	Rate, %		*N*	Median (CI), months	Rate, %	
						12 months	18 months			12 months	18 months
Overall *(N = 205)*	Overall	141	79.4%	201	44.1 (36.3, NR)	84.0%	76.2%	205	44.2 (31.9, NR)	83.5%	73.7%
	1 L → 2 L	47	85.1%	70	43.2 (39.5, NR)	87.5%	80.8%	71	NR (31.9, NR)	86.1%	73.6%
	2 L → 3 L	51	80.4%	70	44.3 (36.3, NR)	86.5%	82.1%	73	44.2 (37.0, NR)	84.5%	78.4%
D_I_ ^*d*^ *(N = 88)*	Overall	60	85.0%	86	NR	87.8%	84.1%	88	NR	84.4%	79.3%
	1 L → 2 L	22	86.4%	35	39.5 (39.5, NR)	92.7%	84.1%	36	39.5 (39.5, NR)	89.7%	77.5%
	2 L → 3 L	26	88.5%	32	NR	89.0%	89.0%	33	NR	87.2%	87.2%
D_p_ ^*d*^ *(N = 76)*	Overall	51	76.5%	74	30.1 (22.1, NR)	82.2%	71.0%	76	30.4 (26.3, NR)	86.2%	75.3%
	1 L → 2 L	10	90.0%	15	31.9 (13.2, NR)	77.8%	62.2%	15	31.9 (12.5, NR)	77.8%	53.3%
	2 L → 3 L	19	68.4%	28	31.8 (22.1, NR)	82.6%	73.1%	30	37.4 (26.3, NR)	84.0%	75.2%
VR *(N = 64)*	Overall	42	71.4%	60	39.5 (31.8, NR)^e^	86.2%	77.0%	64	37.4 (31.6, NR)^e^	82.3%	75.7%
	1 L → 2 L	19	78.9%	30	43.2 (39.5, NR)	88.4%	88.4%	31	NR (39.5, NR)	85.0%	85.0%
	2 L → 3 L	15	73.3%	20	36.3 (23.7, NR)	93.8%	85.9%	23	37.4 (31.6, NR)	85.9%	79.8%

Abbreviations: 1 L, first‐line; 2 L, second‐line; 3 L, third‐line; cBTKi, covalent Bruton tyrosine kinase inhibitor; CI, confidence intervals; CR, complete response; CT, computed tomography; D_I_, discontinuation of prior cBTKi due to intolerance; D_P_, discontinuation of prior cBTKi due to disease progression; NR, not reached; ORR, overall response rate; PFS, progression‐free survival; PR, partial response; TTNT‐D, time to next treatment or death; VR, venetoclax + rituximab.

a PFS was defined as the time from the start of venetoclax‐based therapy to disease progression or death (i.e., event). Patients without events were censored at the last date of follow‐up; medians and corresponding 95% CIs were estimated using the Kaplan–Meier method. Kaplan–Meier estimates at 12 and 18 months were also reported.

b TTNT‐D was defined as the time from the start of venetoclax‐based therapy to alternative treatment or death (i.e., event). Patients without events were censored at the last date of follow‐up; medians and corresponding 95% CIs were estimated using the Kaplan–Meier method. Kaplan–Meier estimates at 12 and 18 months were also reported.

c ORR was measured as the proportion of patients with CR or PR among those with available information on responses in their medical charts based on physician assessment. CR and PR were based on physician‐reported information as recorded in the patient charts. Although CT scans and bone marrow biopsies were not mandated to assess response, abstractors were provided with the 2018 International Workshop on Chronic Lymphocytic Leukemia response criteria for reference when completing data collection.

d Seven patients for whom both intolerance and progression were selected were excluded from the cBTKi discontinuation stratified analyses.

e The longer median PFS than TTNT‐D observed in the VR subgroup may be due to progression events not being systematically reported in the charts for all patients or because progression may not have occurred (in the case of intolerance) prior to initiation of the subsequent treatment in the real‐world data.

*Overall*: ORR was 79.4%, median PFS was 44.1 months (18‐month rate: 76.2%), and median TTNT‐D was 44.2 months (18‐month rate: 73.7%).

*1 L cBTKi➔*2 *L venetoclax group*: ORR was 85.1%, median PFS was 43.2 months (18‐month rate: 80.8%), and median TTNT‐D was not reached (NR; 18‐month rate: 73.6%).

*2 L cBTKi➔*3 *L venetoclax group*: ORR was 80.4%, median PFS was 44.3 months (18‐month rate: 82.1%), and median TTNT‐D was 44.2 months (18‐month rate: 78.4%).

*CT/CIT exposure*: In the *2 L cBTKi➔3 L venetoclax group*, 63/73 (86.3%) patients had exposure to CT/CIT before cBTKi. ORR was 78.6% (among 42/63 [66.7%] patients with response information), median PFS was 44.1 months (18‐month rate: 80.7%), and median TTNT‐D was 44.2 months (18‐month rate: 78.8%). Among the 10 patients without CT/CIT exposure, the ORR was 88.9% (9 [90.0%] patients with response information). PFS and TTNT‐D could not be calculated due to low event rates.

*Overall*: ORR was 85.0%, median PFS was NR (18‐month rate: 84.1%), and median TTNT‐D was NR (18‐month rate: 79.3%).

*1 L cBTKi➔*2 *L venetoclax group*: ORR was 86.4%, median PFS was 39.5 months (18‐month rate: 84.1%), and median TTNT‐D was 39.5 months (18‐month rate: 77.5%).

*2 L cBTKi➔*3 *L venetoclax group*: ORR was 88.5%, median PFS was NR (18‐month rate: 89.0%), and Median TTNT‐D was NR (18‐month rate: 87.2%).

*Overall*: ORR was 76.5%, median PFS was 30.1 months (18‐month rate: 71.0%), and median TTNT‐D was 30.4 months (18‐month rate: 75.3%). The median (IQR) duration on prior cBTKi was 32.0 (14.5, 46.7 months).

*1 L cBTKi➔2 L venetoclax group*: ORR was 90.0%, median PFS was 31.9 months (18‐month rate: 62.2%), and median TTNT‐D was 31.9 months (18‐month rate: 53.3%).

*2 L cBTKi➔3 L venetoclax group*: ORR was 68.4%, median PFS was 31.8 months (18‐month rate: 73.1%), and median TTNT‐D was 37.4 months (18‐month rate: 75.2%).

*Overall*: ORR was 71.4%, median PFS was 39.5 months (18‐month rate: 77.0%), and median TTNT‐D was 37.4 months (18‐month rate: 75.7%).

*1 L cBTKi➔2 L venetoclax group*: ORR was 78.9%, median PFS was 43.2 months (18‐month rate: 88.4%), and median TTNT‐D was NR (18‐month rate: 85.0%).

*2 L cBTKi➔3 L venetoclax group*: ORR was 73.3%, median PFS was 36.3 months (18‐month rate: 85.9%), and median TTNT‐D was 37.4 months (18‐month rate: 79.8%).

This international analysis from the CORE study contributes important, real‐world evidence, showing that patients with CLL initiating venetoclax‐based therapy following cBTKis achieved an ORR of nearly 80%. Moreover, this study is among the first to demonstrate that venetoclax‐based therapy is associated with a long PFS, regardless of its use in the 2 L (43.2 months) or 3 L (44.3 months), even with prior CT/CIT exposure (44.1 months). While this appears to be nearly double the median PFS of 24.7 months reported in the phase 2, open‐label clinical trial of heavily pretreated patients with CLL who relapsed after or were refractory to ibrutinib (M14‐032) [6], the present findings provide real‐world evidence of venetoclax effectiveness following cBTKis, even with CT/CIT pretreatment.

The current study is among the first and the largest to demonstrate high response rates and durable disease control associated with VR among patients previously treated with cBTKis, a finding of particular clinical relevance given the limited prior literature evaluating this real‐world setting and VR's preferable fixed‐duration dosing schedule. Notably, Sobon et al. and Lew et al. assessed the real‐world use of VR in small subgroups of patients with relapsed/refractory CLL and prior cBTKis and observed consistently robust median PFS (25.9–37.0 months) and ORR (81%) [4, 5].

When stratified by reason for cBTKi discontinuation, clinical effectiveness of venetoclax‐based therapy was observed among both those who experienced intolerance (median PFS and TTNT‐D were NR) and those with disease progression following cBTKis (median PFS and TTNT‐D were ~30 months), though outcomes tended to be better in the former group likely because cBTKi progression may indicate more biologically aggressive CLL. A similar trend was observed by Eyre et al. for patients receiving venetoclax monotherapy (after a median of 3 prior lines of therapy), where ORR was slightly higher in the subgroup of patients discontinuing B‐cell receptor inhibitors due to toxicity (92%) rather than CLL progression (84%) [3].

In conclusion, this multicenter, international, real‐world study demonstrates that venetoclax‐based therapy is an effective treatment option with high response rates, both overall in a cohort of older adults and as 2 or 3 L therapy (even with prior CT/CIT exposure) following cBTKi discontinuation due to intolerance or disease progression. Patients receiving fixed‐duration VR as their venetoclax‐based therapy also experienced consistently promising clinical outcomes. Notably, some study limitations should be considered (Supporting Information). As the CLL treatment paradigm continues to evolve, clinicians can use these timely results to inform their clinical practice.

## Ethics Statement

Approval was received from institutional review boards from each of the centers included in the CORE database.

## Consent

Patient consent was not required since the data collected were deidentified and retrospective.

## Conflicts of Interest

NG has received consulting fees from AbbVie, AstraZeneca, Janssen, Bristol Myers Squibb, Gilead Sciences/Kite Pharma, Beigene, Incyte, Roche/Genentech, Novartis, Loxo Oncology, Genmab, ADC Therapeutics and Ascentage Pharma; he has received research funding from Genentech/Roche, Bristol Myers Squibb, Gilead Sciences/Kite Pharma, Incyte, AstraZenca, AbbVie. NL: Advisory board member/consulting for Abbvie, BeiGene, EliLilly/Loxo, Genmab, Janssen, Pharmacyclics, Gilead, AstraZeneca; Institutional Research Funding: AbbVie, AstraZeneca, Eli Lilly/Loxo, Genentech, Genmab, Bei‐Gene, MingSight, Oncternal, Octapharma. TAE: Roche: Education Honorarium, Advisory Board Honorarium, Travel support; Gilead: Honorarium; Research support; travel to scientific conferences; KITE: Advisory Board Honorarium; Janssen: Honorarium; Abbvie: Honorarium; travel to scientific conferences; AstraZeneca: Honorarium, Research funding; Loxo Oncology: Advisory Board Honorarium, Trial steering committee; Beigene: Advisory Board Honorarium, Research funding; Incyte: Advisory Board Honorarium; Secura Bio: Advisory Board Honorarium. CCC: Received honoraria/consulted for AbbVie, Allogene, AstraZeneca, Beigene, Genentech, Janssen, LOXO/Lilly, MEI Pharma, Mingsight, Octapharma, TG Therapeutics; Speaker's Bureau: AbbVie, AstraZeneca, Beigene, Genentech; Received research funding (paid to the institution) from AbbVie, AstraZeneca, Beigene, CarnaBio, LOXO/Lilly. BSM, NE, DJ, WS: employees of AbbVie and may hold stock or stock options. SEM: retired from AbbVie. JRB: Consultancy: Abbvie, Acerta/Astra‐Zeneca, Alloplex Biotherapeutics, BeiGene, Galapagos NV, Genentech/Roche, Grifols Worldwide Operations, InnoCare Pharma Inc. iOnctura, Kite, Loxo/Lilly, Merck, Numab Therapeutics, Pfizer, Pharmacyclics; Research funding: BeiGene, Gilead, iOnctura, Loxo/Lilly, MEI Pharma, TG Therapeutics. HHT: Consultancy: Abbvie; Advisory Board: EliLilly/LOXO and Astrazeneca CU: Honorarium—Abbvie, Astrazeneca, Beigene, Genentech, Jansen, Lilly, Pharmacyclics; Research funding—AbbVie, Astrazeneca, Lilly, PCYC. LAL: Kite/Gilead, Beigene, Pharmacyclics, Abbvie, Genmab, SeaGen, Janssen, AstraZeneca, Eli Lily, Epizyme. Consultant/ad board: ADC Therapeutics, Kite/Gilead, Beigene, Pharmacyclics, Abbvie, Genmab, SeaGen, Janssen, AstraZeneca, Eli Lily, Epizyme, Merck. LER: Consultant for AbbVie, Ascentage, AstraZeneca, Beigene, Janssen, Loxo Oncology, Pharmacyclics, Pfizer, TG Therapeutics, is a member of a data safety monitoring committee (DSMC) for Ascentage, served as a CME speaker for DAVA, Curio, Medscape, and PeerView, holds minority ownership interest in Abbott Laboratories, received travel support from LOXO oncology, and has received research funding (paid to the institution) from Adaptive Biotechnologies, AstraZeneca, Genentech, AbbVie, Pfizer, Loxo Oncology, Aptose Biosciences, Dren Bio, and Qilu Puget Sound Biotherapeutics. LER is also supported in part by National Institutes of Health/National Cancer Institute Cancer Center support grant P30 CA008748. MSD: received research funding from AbbVie, AstraZeneca, Ascentage Pharma, Genentech, Novartis, Surface Oncology; and consulting fees from AbbVie, Adaptive Biosciences, Ascentage Pharma, AstraZeneca, BeiGene, BMS, Eli Lilly, Genentech, Genmab, Janssen, Merck, Mingsight Pharmaceuticals, Nuvalent, ONO Pharmaceuticals, Secura Bio, TG Therapeutics, Takeda. JMR: Consultancy: Abbvie, AstraZeneca, ADC Therapeutics, Beigene, Epizyme, Genentech, GenMab, Loxo Oncology, Janssen, Morphosys, Pharmacyclics, Pfizer Membership on a Board or Advisory Committee: Abbvie, Beigene, Janssen, Research Funding: Acerta, Beigene, Epizyme, Janssen, Loxo Oncology, Oncternal, Pharmacyclics, Velosbios. APS: Research support: AbbVie, Pharmacyclics, Verastem, Acerta Pharma, AstraZeneca, Celgene, Kite Pharma, and Bristol‐Myers Squibb; Honoraria: AbbVie, Pharmacyclics, Genentech, Seattle Genetics, Novartis, Verastem, Jazz Pharmaceuticals, AstraZeneca, Celgene, Kite Pharma, BeiGene, Gilead Sciences; Consultancy: AbbVie, Pharmacyclics, AstraZeneca, Kite Pharma, Celgene, Genentech, Jazz Pharmaceuticals, Alexion; Speaker and consulting fees: Lilly, ADC Therapeutics. IF: Consultant and/or Advisory board: Abbvie, Astrazeneca, Beigene, Bristol Myers Squibb, Gilead, Incyte Janssen, Merck, Novartis, Roche, Seattle Genetics. BTH: Advisory boards for Genentech, AbbVie, Pharmacyclics, Gilead. Research funding: Genentech, AbbVie, Pharmacyclics. NM: Speaker and travel support: AbbVie, AstraZeneca, Janssen; Advisory Boards: AbbVie, AstraZeneca, BeiGene, Takeda. PMB: Consultancy for AbbVie; Research funding: Astrazeneca, TG therapeutics; consulting for AstraZeneca, TG, Pharmacyclics, Celgene, Genentech, Morphosys, Seattle Genetics, MEI, Gilead, Janssen. LP: Nothing to disclose. FL: Nothing to disclose. YC: Nothing to disclose. CEJ: Consultancy: AbbVie. SJS: Consultant and Institutional Research Funding: Novartis, Pharmacyclics, Celgene; Institutional Research Funding: Gilead, Janssen Research & Development, Merck; Scientific Advisory Committees: Nordic Nanovector; Consultant: Genentech, Acerta. IP, TW, and AG are employees of Analysis Group Inc. which has received consultancy fees from AbbVie. BF: Consultancy and Advisory Board: AbbVie, ADC therapeutics, AstraZeneca, BeiGene, BMS, Eli Lilly, Genentech, Genmab, Pharmacyclics; Research funding: AbbVie/Genmab, Loxo‐Lilly, BMS, Angiocrine. Speaker Bureau: Loxo/Lilly. MS: Consulting, advisory boards, steering committees or data safety monitoring committees: Abbvie, Genentech, AstraZeneca, Genmab, Janssen, Beigene, Bristol Myers Squibb, Morphosys/Incyte, Kite Pharma, Eli Lilly, Mustang Bio, Fate therapeutics, Nurix and Merck; institutional research funding from Mustang Bio, Genentech, AbbVie, Beigene, AstraZeneca, Genmab, Morphosys/Incyte, and Vincerx; Stock options: Koi Biotherapeutics; Employment: Bristol Myers Squibb (spouse). DMS: has received consulting fees from Abbvie, AstraZeneca, Beigene, BMS, Celegene, Eli Lilly, Genentech, Janssen, Pharmacyclics. My institution has received clinical research grants from AstraZeneca, Novartis, BMS. MC: Nothing to disclose.

## Supporting information


Data S1.

## Data Availability

The data analyzed in this study are subject to Health Insurance Portability and Accountability Act privacy restrictions and are not publicly available. Deidentified data could be made available by the corresponding author upon reasonable request. AbbVie is committed to responsible data sharing regarding the clinical trials we sponsor. This includes access to anonymized, individual, and trial‐level data (analysis data sets), as well as other information (e.g., protocols, clinical study reports, or analysis plans), as long as the trials are not part of an ongoing or planned regulatory submission. This includes requests for clinical trial data for unlicensed products and indications. These clinical trial data can be requested by any qualified researchers, who engage in rigorous, independent, scientific research, and will be provided following review and approval of a research proposal, Statistical Analysis Plan (SAP), and execution of a Data Sharing Agreement (DSA). Data requests can be submitted at any time after approval in the US and Europe and after acceptance of this manuscript for publication. The data will be accessible for 12 months, with possible extensions considered. For more information on the process or to submit a request, visit the following link: https://vivli.org/ourmember/abbvie/ then select “Home”.
