# Supplementary material for: Treatment Effectiveness of Venetoclax‐Based Therapy After Bruton Tyrosine Kinase Inhibitors in Chronic Lymphocytic Leukemia: An International Real‐World Study
Source: Am J Hematol. 2024 Dec 19;100(3):511–5. doi: 10.1002/ajh.27563 (PMC11803543; doi:10.1002/ajh.27563)
Supplement: Supplementary file 1 — Data S1. [file AJH-100-511-s001.docx]

# Supplementary material

## Supplementary methods

### Data source

CORE is an ongoing, retrospective, observational study of patients from 23 medical centers in the US, Canada, and Europe. The study collects continuous longitudinal data on patients with chronic lymphocytic leukemia (CLL) from the participating sites, using a chart review approach to capture information at regular intervals on patient demographics, clinical characteristics, comorbidities, the number and types of treatments patients received, physician-reported outcomes, and management after disease progression (if it occurs). Ethics approval was received from institutional review boards from each of the centers included in this study prior to study commencement.

### Study measures and analyses

Baseline characteristics at initiation of venetoclax-based therapy and treatment characteristics of venetoclax-based therapy and the prior covalent Bruton tyrosine kinase inhibitor (cBTKi) were summarized descriptively. Mean, median, standard deviation (SD), and interquartile range (IQR) were used to summarize continuous variables, while counts and percentages were used to summarize categorical variables.

Overall response rate (ORR) was measured as the proportion of patients with complete response (CR) or partial response (PR) among those with available information on responses in their medical charts based on physician assessment. CR and PR were based on physician-reported information as recorded in the patient charts. Although computed tomography (CT) scans and bone marrow biopsies were not mandated to assess response, abstractors were provided with the 2018 International Workshop on Chronic Lymphocytic Leukemia (iwCLL) response criteria for reference when completing data collection.^1^ Progression-free survival (PFS) was defined as the time from the start of venetoclax-based therapy to disease progression or death (i.e., event). Time to next treatment or death (TTNT-D) was defined as the time from the start of venetoclax-based therapy to alternative treatment or death (i.e., event). For both PFS and TTNT-D, patients without events were censored at the last date of follow-up; medians and corresponding 95% confidence intervals (CIs) were estimated using the Kaplan-Meier method. Kaplan-Meier estimates at 12 and 18 months were also reported.

Seven patients for whom both intolerance and progression were selected were excluded from the cBTKi discontinuation stratified analyses.

## Supplementary results

### Patient characteristics

Among the 205 patients in the study, 40.7% initiated their first-line (1L) therapy prior to 2014. Rai staging was equally distributed between stage 0-II (51.0%) and III-IV (49.0%) among patients who were assessed. The proportion of patients with unmutated IGHV was highest among patients who discontinued due to progression (discontinuation of prior cBTKi due to intolerance [D_I_]: 63.0% [17/27], discontinuation of prior cBTKi due to disease progression [D_P_]: 73.1% [19/26], venetoclax + rituximab [VR]: 69.6% [16/23]), as was the proportion of patients with 17p deletion or TP53 mutation (D_I_: 14.3% [7/49], D_P_: 34.6% [18/52], VR: 17.4% [8/46]). The most common comorbidities for all patients were cardiovascular (46.8%) and endocrine/metabolic (23.4%) conditions.

### Treatment characteristics

A total of 49.3% of patients (101/205) remained on treatment at analysis, 12.2% (25/205) completed their scheduled therapy duration, and 38.5% (79/205) ended therapy for other reasons, with the most common being intolerance (31.6%) and disease progression (17.7%). Among patients treated with VR, 39.1% (25/64) remained on treatment at analysis, 26.6% (17/64) completed their scheduled therapy duration, and 34.4% (22/64) ended therapy for other reasons, with the most common being intolerance (14.0% [9/64]) and disease progression (7.8% [5/64]).

Median (IQR) time from cBTKi discontinuation to venetoclax-based therapy initiation was 1.9 (0.3, 11.0) months for D_I_ patients and 0.1 (0.0, 0.9) months for D_P_ patients.

## Supplementary limitations

The results of this study should be interpreted within the context of certain limitations. Since data collection was based on information recorded in patients’ charts, there may have been data entry differences or errors across centers. Relatedly, while data abstractors were provided with the iwCLL criteria for reference, treatment response information was ultimately based on physicians’ assessment and thus may have varied across centers as CT scans/bone marrow evaluations were not uniformly done to characterize response. Additionally, information on minimal residual disease was not available in the medical records. The smaller sample size precluded additional statistical analysis of the potential impacts of treatment factors, such as type of venetoclax-based therapy and fixed duration dosing. The number of patients receiving VR as their venetoclax-based therapy may have been potentially impacted due to our data collection period overlapping with the COVID-19 pandemic, where anti-CD20 therapy was found to be associated with an increased risk of severe COVID-19 outcomes.^2^ The most common cBTKi observed was ibrutinib-based regimens which may limit generalizability of the findings to other BTKis. Lastly, the duration of follow-up was relatively short, and since patients had variable follow-up, not all progression events may have been accrued or reported; additional analyses with longer follow-up and duration of venetoclax therapy are warranted. Further analysis of this study with larger cohorts and longer follow-up time will be undertaken as part of future work to grow this body of evidence.

## References

1. Hallek M, Cheson BD, Catovsky D, et al. iwCLL guidelines for diagnosis, indications for treatment, response assessment, and supportive management of CLL. *Blood*. 2018;131(25):2745-2760.
2. Boekel L, Wolbink GJ. Rituximab during the COVID-19 pandemic: time to discuss treatment options with patients. *Lancet Rheumatol.* 2022;4(3):e154-e155.

**Supplementary Table 1. Patient demographics and clinical characteristics at time of initiation of venetoclax-based therapy**

| **Patient characteristics** | **Overall (N=205)** |
| --- | --- |
| **Age at venetoclax-based therapy initiation (years), mean ± SD [median] (IQR)** | 68.7 ± 10.4 [68.7] (62.2, 76.4) |
| **Male sex, N (%)** | 141 (68.8%) |
| **Practice setting, N (%)** |  |
| Academic | 196 (95.6%) |
| Community | 9 (4.4%) |
| **Most common insurance types, N (%)^1^** |  |
| Medicare | 86 (42.0) |
| Commercial/private insurance | 69 (33.7%) |
| **Rai staging assessed, N (%)^2^** | 149 (72.7) |
| Stage 0 - II | 76 (51.0) |
| Stage III – IV | 73 (49.0) |
| **ECOG assessed, N (%)^2^** | 156 (76.1) |
| Grade 0 | 55 (35.3) |
| Grade 1 – 2 | 96 (61.5) |
| Grade 3 – 4^3^ | 5 (3.2) |
| **Risk of TLS assessed, N (%)^2^** | 170 (82.9) |
| Low | 85 (50.0) |
| Medium | 57 (33.5) |
| High | 28 (16.5) |
| ***IGHV* status assessed, N (%)^2^** | 69 (33.7) |
| Unmutated | 48 (69.6) |
| **Chromosomal abnormalities assessed, N (%)^2^** | 124 (60.5) |
| 13q deletion or *LAMP1* mutation | 42 (33.9) |
| 17p deletion or *TP53* mutation | 30 (24.2) |
| Trisomy 12 | 24 (19.4) |
| 11q deletion of *ATM* mutation | 26 (21.0%) |
| **Genetic mutations assessed, N (%)^2^** | 84 (41.0%) |
| *TP53* | 21 (25.0) |
| *NOTCH1* | 11 (13.1) |
| *BTK/PLCG2 (ibrutinib resistance)* | 6 (7.1) |
| **Total number of comorbidities, mean ± SD [median] (IQR)** | 1.4 ± 1.3 [1.0] (0.0, 2.0) |
| **Most common comorbidities, N (%)^1^** |  |
| Cardiovascular | 96 (46.8%) |
| Endocrine/metabolic | 48 (23.4%) |
| Renal | 28 (13.7%) |
| Other clinically significant condition | 31 (15.1%) |
| Respiratory | 29 (14.1%) |
| Neurological | 20 (9.8%) |

Abbreviations: ECOG: Eastern Cooperative Oncology Group; IGVH: immunoglobulin heavy-chain variable region gene; IQR: interquartile range; SD: standard deviation; TLS: tumor lysis syndrome.

1. Insurance categories are not mutually exclusive, with the exception of patients for whom no insurance or unknown insurance status was indicated. Additional categories with prevalences of ≤5.9% included Medicaid, public insurance plan, private insurance plan, military insurance, and no insurance, with 25.9% with unknown insurance status.

2. Percentages are calculated out of patients with available data.

3. One patient had ECOG Grade 4.

**Supplementary Table 2. Treatment characteristics of venetoclax-based therapy following discontinuation of cBTKi**

| **Treatment characteristics** | **Overall** |
| --- | --- |
|  | **(N=205)** |
| **Time from diagnosis to venetoclax-based therapy initiation (months), mean ± SD [median] (IQR)** | 89.4 ± 65.8 [77.4] (34.7, 124.5) |
| **Time from venetoclax-based therapy initiation to end of follow-up (months), mean ± SD [median] (IQR)** | 20.1 ± 16.5 [16.5] (6.0, 30.5) |
| **Venetoclax-based therapy treatment duration (months), mean ± SD [median] (IQR)** | 17.8 ± 15.1 [14.4] (3.9, 28.0) |
| **Time from discontinuation of cBTKi to initiation of venetoclax (months), mean ± SD [median] (IQR)** | 4.4 ± 9.5 [0.6] (0.0, 3.8) |
| Among patients with intolerance | 7.3 ± 11.7 [1.9] (0.3, 11.0) |
| Among patients with progression | 2.0 ± 7.4 [0.1] (0.0, 0.9) |
| **Prior cBTKi treatment duration (months), mean ± SD [median] (IQR)** | 24.9 ± 19.9 [20.2] (8.4, 39.2) |
| cBTKi discontinuation due to intolerance^1^ | 21.2 ± 19.5 [16.5] (4.0, 32.0) |
| cBTKi discontinuation due to progression^1^ | 32.2 ± 20.2 [32.0] (14.5, 46.7) |
| **Type of prior cBTKi therapy received, N (%)** |  |
| Ibrutinib | 175 (85.4%) |
| Acalabrutinib | 14 (6.8%) |
| Ibrutinib + rituximab | 6 (2.9%) |
| Zanubrutinib | 4 (2.0%) |
| Other^2^ | 6 (2.9%) |
| **Type of venetoclax-based therapy received, N (%)** |  |
| Venetoclax monotherapy | 123 (60.0) |
| Venetoclax combination therapy | 82 (40.0) |
| VR | 64 (78.0) |
| VO | 18 (22.0) |
| **Line of therapy in which venetoclax-based therapy was initiated, N (%)** |  |
| 2L^3^ | 71 (34.6%) |
| 3L^4^ | 73 (35.6%) |
| 4L+^5^ | 61 (29.8%) |
| **Dose changes of venetoclax-based therapy, N (%)** |  |
| Maintained | 164 (80.0%) |
| Reduced | 37 (18.0%) |
| Unknown | 4 (2.0) |
| **Primary reason for discontinuation of prior cBTKi, N (%)^6^** |  |
| Intolerance | 88 (42.9) |
| Disease progression | 76 (37.1) |
| Patient completed scheduled duration of therapy | 14 (6.8) |
| Patient’s request | 10 (4.9) |

Abbreviations: 2L: second-line; 3L: third-line; 4L+: fourth-line or later; cBTKi: covalent Bruton tyrosine kinase inhibitor; IQR: interquartile range; SD: standard deviation; VO: venetoclax + obinutuzumab; VR: venetoclax + rituximab.

1. Seven patients who discontinued due to both intolerance and progression were excluded.

2. Other regimens included ibrutinib + bendamustine + obinutuzumab, ibrutinib + BNC105P, ibrutinib + cyclophosphamide + vincristine, ibrutinib + ofatumumab, ibrutinib + rituximab + ifosfamide + carboplastin + etoposide, and ibrutinib + umbralisib.

3. In the second line, 33 patients initiated venetoclax monotherapy, and 38 patients initiated venetoclax combination therapy (with rituximab: 31/71 [43.7%]; with obinutuzumab: 7/71 [9.9%])

4. In the third line, 42 patients initiated venetoclax monotherapy, and 31 patients initiated venetoclax combination therapy (with rituximab: 23/73 [31.5%]; with obinutuzumab: 8/73 [11.0%])

5. In the fourth line or later, 48 patients initiated venetoclax monotherapy, and 13 patients initiated venetoclax combination therapy (with rituximab: 10/62 [16.1%]; with obinutuzumab: 3/62 [4.8%])

6. Categories are mutually exclusive; patients who reported both intolerance and disease progression as reasons for discontinuation were excluded from these counts.

**Supplementary Figure 1. Sample selection**

|  | All patients  **N=2,293** |  |  |  |
| --- | --- | --- | --- | --- |
|  |  |  |  |  |
| Did not initiate a cBTKi in any line of therapy  **N=749 (32.6%)** |  | Initiated a cBTKi in ≥1 line of therapy  **N=1,544 (67.4%)** |  |  |
|  |  |  |  |  |
|  |  | Discontinued cBTKi  **N=718 (46.5%)** |  |  |
|  |  |  |  |  |
|  |  | Initiated a subsequent line of therapy^1^  **N=487 (67.8%)** |  |  |
|  |  |  |  |  |
|  |  | cBTKi 🡪 venetoclax  **N=205 (42.1%)** |  |  |
|  |  |  |  |  |
|  | cBTKi 🡪 venetoclax monotherapy  **N=123 (60.0%)** |  | cBTKi 🡪 venetoclax combination therapy  **N=82 (40.0%)** |  |
|  |  |  |  |  |
|  |  | cBTKi 🡪 VR  **N=64 (78.0%)** |  | cBTKi 🡪 VO  **N=18 (22.0%)** |
|  |  |  |  |  |

Abbreviations: cBTKi: covalent Bruton tyrosine kinase inhibitor; VO: venetoclax + obinutuzumab; VR: venetoclax + rituximab.

1. Among the 487 patients who initiated a subsequent line, 282 initiated a non-venetoclax-based therapy, mostly another cBTKi (33.7%; e.g., acalabrutinib, ibrutinib) or CT/CIT (19.1%).

**Supplementary Figure 2. ORR for the overall cohort and stratified by line of therapy, by reason for cBTKi discontinuation, and for patients treated with VR^1^**


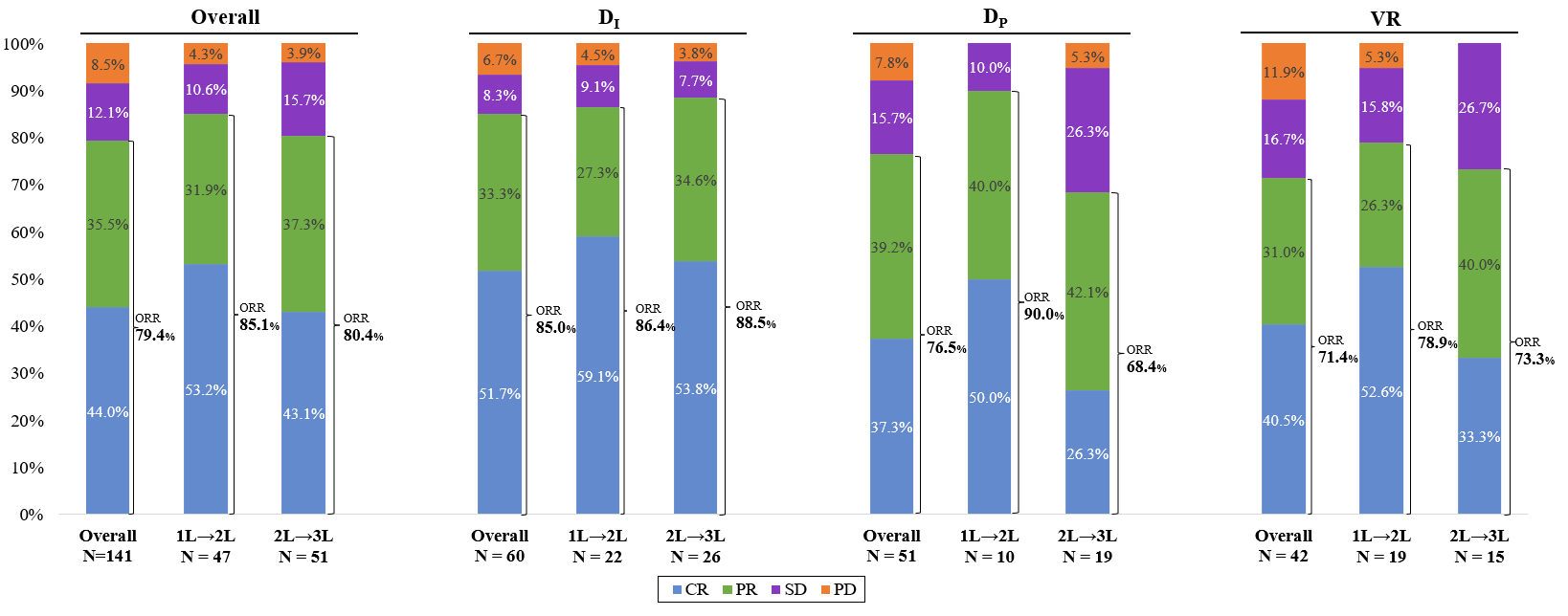


Abbreviations: 1L: first-line; 2L: second-line; 3L: third-line; CR: complete response; D_I_: discontinuation of prior cBTKi due to intolerance; D_P_: discontinuation of prior cBTKi due to disease progression; ORR: overall response rate; PD: progressive disease; PR: partial response; SD: stable disease; VR: venetoclax + rituximab.

1. Percentages were calculated among patients with a documented response.

**Supplementary Figure 3. PFS for the overall cohort and stratified by line of therapy, by reason for cBTKi discontinuation, and for patients treated with VR^1-3^**

**
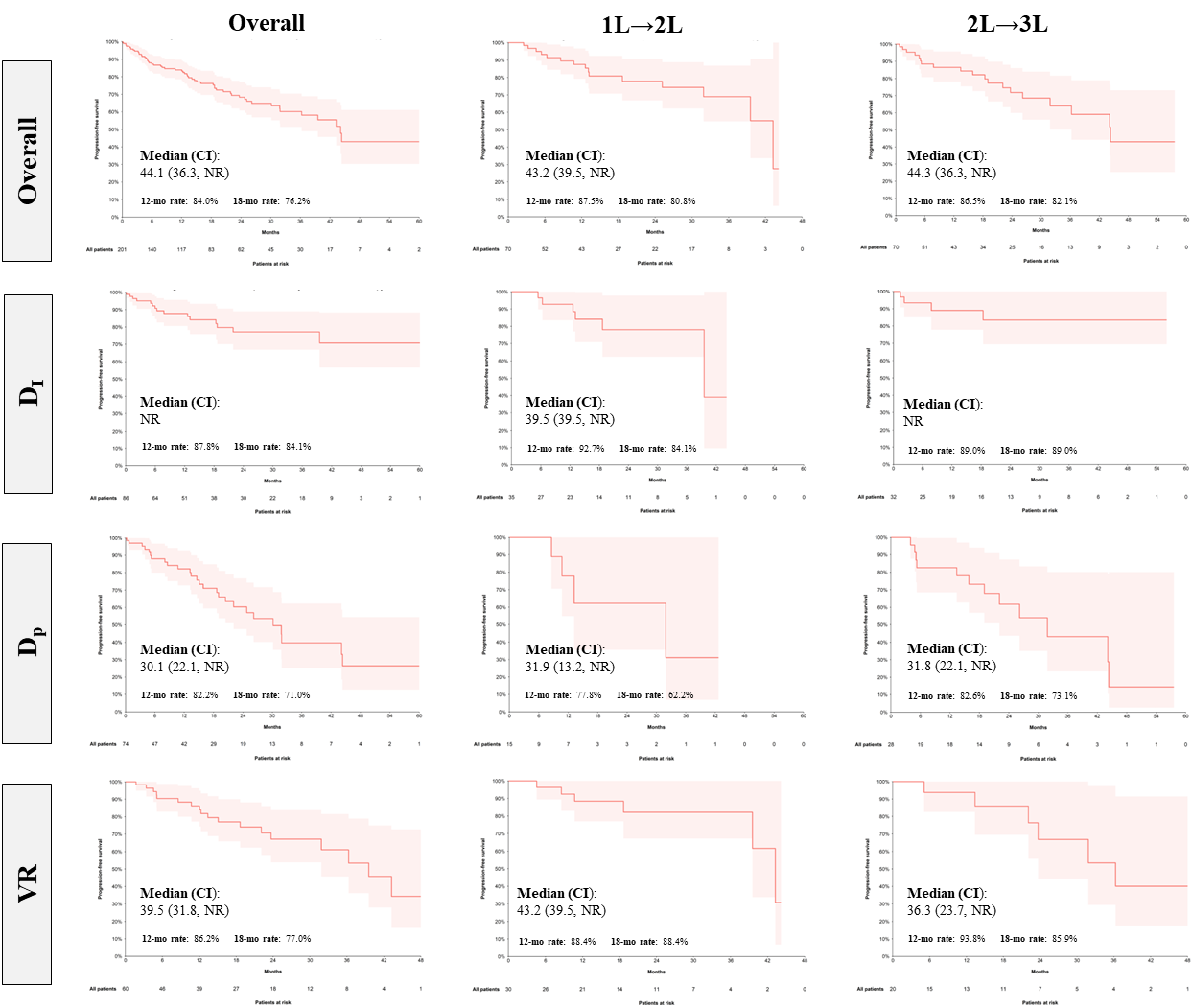
**

Abbreviations: 1L: first-line; 2L: second-line; 3L: third-line; CI: confidence interval; D_I_: discontinuation of prior cBTKi due to intolerance; D_P_: discontinuation of prior cBTKi due to disease progression; NR: not reached; PFS: progression-free survival; VR: venetoclax + rituximab.

1. PFS was measured from date of initiation to disease progression or death (event), or end of follow-up period.

2. Four patients were excluded due to missing time-to-event information, including one patient who received venetoclax-based therapy in the 1L→2L (*by subgroup*: D_I_: 1 patient; D_P_: 0 patients; VR: 1 patient), and three patients who received venetoclax-based therapy in the 2L→3L (*by subgroup*: D_I_: 1 patient; D_P_: 2 patients; VR: 3 patients).

3. Within the overall study sample, 56/201 patients with available time-to-event data had an event across any line (1L cBTKi→2L venetoclax: 15/70 patients; 2L cBTKi→3L venetoclax: 19/70 patients).

**Supplementary Figure 4. TTNT-D for the overall cohort and stratified by line of therapy, by reason for cBTKi discontinuation, and for patients treated with VR^1^**

**
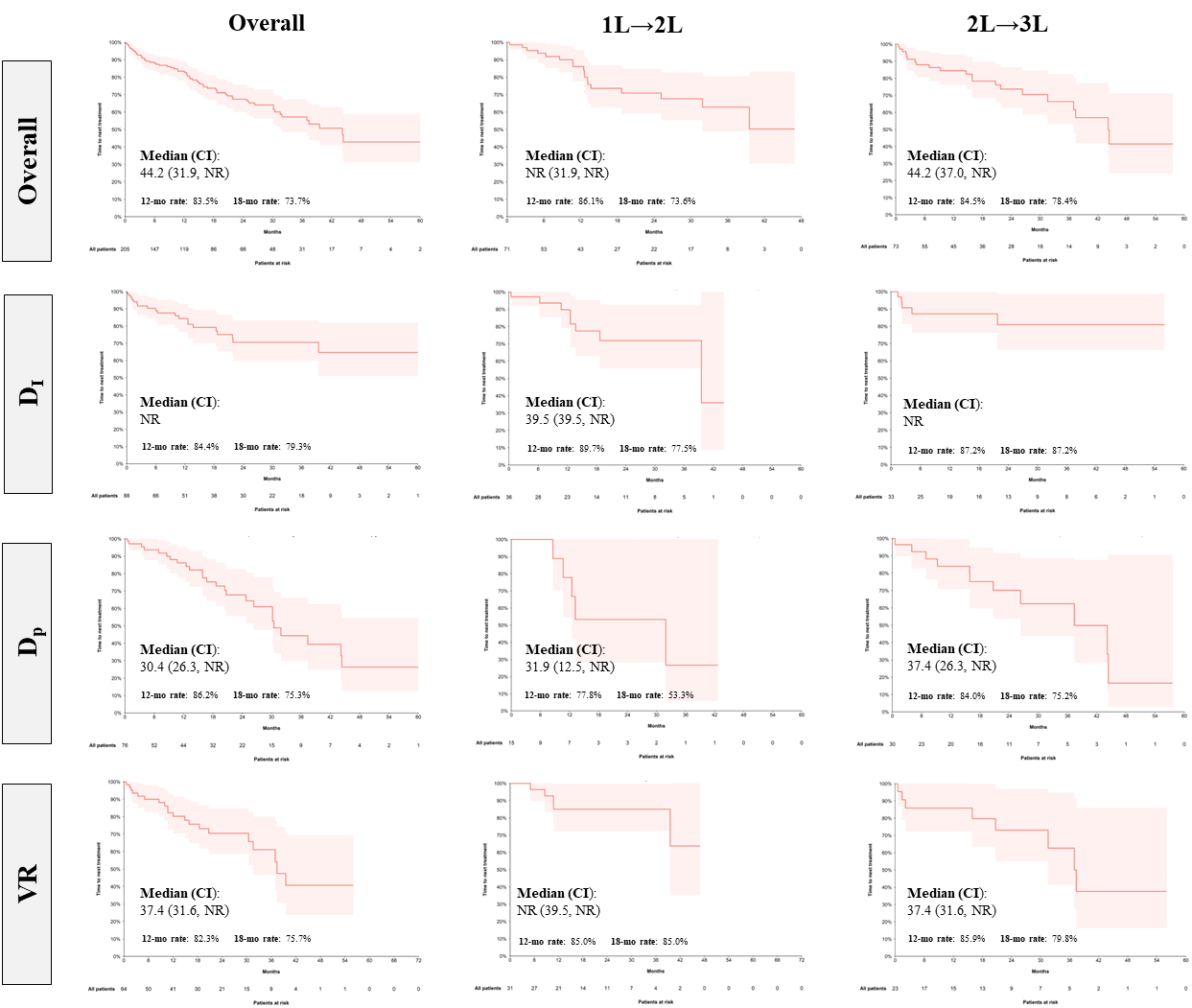
**

Abbreviations: 1L: first-line; 2L: second-line; 3L: third-line; CI: confidence interval; D_I_: discontinuation of prior cBTKi due to intolerance; D_P_: discontinuation of prior cBTKi due to disease progression; NR: not reached; TTNT-D: time to next treatment or death; VR: venetoclax + rituximab.

1. TTNT-D was measured from date of initiation to disease progression or death (event), or end of follow-up period.
